# Dynamics of Physicochemical Properties, Flavor, and Bioactive Components in *Lactobacillus*-Fermented *Pueraria lobata* with Potential Hypolipidemic Mechanisms

**DOI:** 10.3390/foods14193425

**Published:** 2025-10-05

**Authors:** Ye Tang, Liqin Li, Qiong Li, Zhe Li, Huanhuan Dong, Hua Zhang, Huaping Pan, Weifeng Zhu, Zhenzhong Zang, Yongmei Guan

**Affiliations:** 1 Key Laboratory of Modern Preparation of Traditional Chinese Medicine, Ministry of Education, Jiangxi University of Chinese Medicine, Nanchang 330004, China; tangye200111@126.com (Y.T.); 18270150860@163.com (L.L.); zwf0322@126.com (W.Z.); 2 School of Pharmacy, Jiangxi University of Chinese Medicine, Nanchang 330004, China; jzliqiong@163.com (Q.L.); lizhezd@163.com (Z.L.); donghh@jxutcm.edu.cn (H.D.); 20191002@jxutcm.edu.cn (H.Z.); huaping@jxutcm.edu.cn (H.P.); 3 National Key Laboratory of Classic Formula Modern Chinese Medicine Creation, Nanchang 330004, China

**Keywords:** active ingredients, hypolipidemic, *Lactobacillus* fermentation, network pharmacology, *Pueraria lobata*

## Abstract

This study systematically analyzed the multidimensional effects of *Lactobacillus* fermentation on *Pueraria lobata* (*PL*) and investigated the potential mechanisms underlying its hypolipidemic activity. Results indicated that fermentation significantly increased the total acid content from 1.02 to 3.48 g·L^−1^, representing a 2.41-fold increase. Although slight reductions were observed in total flavonoids (8.67%) and total phenolics (6.72%), the majority of bioactive components were well preserved. Other antioxidant capacities were retained at >74.71% of baseline, except hydroxyl radical scavenging. Flavor profiling showed increased sourness and astringency, accompanied by reduced bitterness, with volatile compounds such as β-pinene and trans-2-hexenyl butyrate contributing to a distinct aromatic profile. Untargeted metabolomics analysis revealed that fermentation specifically enhanced the abundance of low-concentration isoflavone aglycones, including daidzein and genistein, suggesting a compositional shift that may improve hypolipidemic efficacy. Integrated network pharmacology and computational modeling predicted that eight key components, including genistein, could stably bind to ten core targets (e.g., AKT1 and MMP9) primarily through hydrogen bonding and hydrophobic interactions, potentially regulating lipid metabolism via the PI3K-AKT, PPAR, and estrogen signaling pathways. This study reveals the role of *Lactobacillus* fermentation in promoting the conversion of isoflavone glycosides to aglycones in *PL* and constructs a multi-dimensional “components-targets-pathways-disease” network, providing both experimental evidence and a theoretical foundation for further research on the lipid-lowering mechanisms of fermented *PL* and the development of related functional products.

## 1. Introduction

Amid global economic growth and dietary shifts, dyslipidemia has emerged as a major public health concern, significantly contributing to the prevalence of cardiovascular and metabolic diseases such as atherosclerosis and fatty liver disease [1]. *Pueraria lobata* (Willd) Ohwi (Gegen), the dried root of a plant in the *Pueraria* genus, is a traditional medicinal and edible resource. It is not only a source of pharmacologically active isoflavonoids like puerarin and daidzin but also rich in nutrients such as polysaccharides and amino acids, embodying the principles of both traditional Chinese medicine and modern nutritional science [2]. Previous studies have demonstrated that *PL* supplementation significantly reduces serum levels of total cholesterol (TC), triglycerides (TG), and low-density lipoprotein cholesterol (LDL-C), while increasing high-density lipoprotein cholesterol (HDL-C) in individuals with dyslipidemia, highlighting its strong lipid-lowering potential [3]. These findings underscore the value of *PL* for developing functional foods aimed at improving blood lipid profiles.

In recent years, microbial fermentation technology has gained increasing attention for its potential to enhance the content of bioactive components and improve the efficacy of herbal materials [4]. Among various microorganisms, particularly *Lactobacillus* strains, have become a preferred choice for fermentation due to their recognized probiotic properties, safety, and versatile enzymatic activities [5]. For instance, Huang [6] reported that fermentation of *PL* by *Lactobacillus reuteri* substantially increased its inhibitory activity against xanthine oxidase (XOD), an effect attributed to the enrichment of specific compounds such as puerarin xyloside and kaempferol-3-rhamnoside-4′-xyloside. In another study, Zhao [7] observed that fermented *PL* products elevated the activities of catalase and superoxide dismutase in rats with alcohol-induced liver injury, reduced lipid accumulation and inflammation, and ultimately ameliorated hepatic damage. Furthermore, Zhong [8] demonstrated that probiotic-fermented blueberry juice inhibited α-glucosidase and α-amylase activities and enhanced glucose consumption in HepG2 cells, indicating its anti-diabetic potential. These collective findings suggest that *Lactobacillus* fermentation holds promise for enhancing the lipid-lowering efficacy of *PL*.

It is noteworthy that different *Lactobacillus* strains exhibit distinct, strain-specific properties and bioconversion capabilities during the fermentation of *PL* [9]. Our prior research identified *Lactobacillus rhamnosus* and *Lactiplantibacillus plantarum P9* as promising candidates due to their excellent probiotic and fermentation characteristics in a *PL*-based matrix [10]. Nevertheless, the dynamic changes in the physicochemical properties, flavor profile, and broad-spectrum bioactive components throughout the fermentation process remain insufficiently explored. Moreover, current research on *PL* fermentation often reports changes in active components and efficacy evaluations in isolation, failing to establish a clear mechanistic link between compositional shifts and therapeutic outcomes, which limits the development of high-quality functional *PL* products.

Advances in analytical technologies provide powerful tools to address these gaps. Intelligent sensory technology, combined with liquid chromatography-mass spectrometry (LC-MS), has emerged as a key approach for elucidating flavor evolution and the transformation of bioactive components during fermentation [11]. Concurrently, network pharmacology, a systems biology-based method, can construct multi-dimensional “component-target-pathway” networks to elucidate the synergistic mechanisms of traditional Chinese medicines. Furthermore, molecular docking (MD) and molecular dynamics simulations (MDS) can predict the binding stability and interactions between active constituents and potential targets at the atomic level, providing computational evidence for the hypothesized lipid-lowering mechanisms of fermented *PL* [12].

In summary, building upon our team’s previous findings, this study employs a comprehensive strategy that integrates intelligent sensory analysis, UHPLC-Q-TOF/MS-based metabolomics, network pharmacology, MD, and MDS. The objective is to systematically investigate the flavor changes, map the transformation patterns of active components, and predict the potential lipid-lowering mechanisms of *Lactobacillus*-fermented *PL*. This research aims to provide a solid experimental foundation for further elucidating the mechanisms of action of *PL* fermentation products and to offer a theoretical basis and a research paradigm for developing fermented *PL*-based health products for lipid management.

## 2. Materials and Methods

### 2.1. Chemicals and Instruments

*Pueraria lobata* (Batch No: 20240301) were purchased from Jiangxi Hongbang Chinese Herbal Medicine Co., Ltd., located in Zhangshu City, China. The puerarin content in the dried *Pueraria lobata* decoction pieces was determined to be ≥2.4% by both the manufacturer and our research group, meeting the requirement of the *Chinese Pharmacopoeia*. *Lactobacillus rhamnosus* HCS01-013 was provided by Jiangxi Renren Health Microbial Resource Library, while *Lactobacillus plantarum* P9 was supplied by China Resources Jiangzhong Pharmaceutical Group Co., Ltd. (Nanchang, China). Standards including gallic acid, puerarin, and glucose had purities greater than 98%, purchased from Chengdu Pufei Bio-Technology Co., Ltd. (Chengdu, China). Folin-Phenol, DNS (3,5-dinitrosalicylic acid), DPPH (1,1-diphenyl-2-trinitrophenylhydrazine), Trolox (water soluble vitamin E), and potassium peroxydisulfate were obtained from Shanghai Yuanye Biotechnology Co., Ltd. (Shanghai, China). ABTS ((2,2′-Azino-bis (3-ethylbenzothiazoline-6-sulfonic acid)), TPTZ (2,4,6-Tri (2-pyridyl)-s-triazine) and sodium acetate trihydrate were sourced from Shanghai Yine Chemical Technology Co., Ltd. (Shanghai, China). Sodium carbonate anhydrous, ferrous sulfate heptahydrate and formic acid were purchased from Shanghai Aladdin Biochemical Technology Co., Ltd. (Shanghai, China). Mass spectrometry grade acetonitrile was obtained from Merck & Co., Inc. (Darmstadt, Germany).

UV-2550 ultraviolet-visible spectrophotometer (Shimadzu Corporation, Kyoto, Japan); TECAN Spark multifunctional microplate reader (TECAN Experimental Equipment Co., Ltd., Shanghai, China); SuperNose electronic nose system (Shanghai Ruiben International Trading Co., Ltd., Shanghai, China); SA-402B electronic tongue instrument (Insent Inc., Aichi, Japan); Heracles NEO ultra-fast gas chromatography electronic nose system (Alpha MOS, Toulouse, France); Triple TOF 5600 Plus high-resolution mass spectrometer (Sciex Corporation, Framingham, MA, USA); LC-30A ultra-high performance liquid chromatography system (Shimadzu Corporation, Kyoto, Japan).

### 2.2. Sample Preparation of PL Fermentation Products

*PL* decoction pieces were pulverized using a mechanical grinder and sieved through a No. 3 Pharmacopoeia sieve for uniform particle distribution. A predetermined amount of powdered material was resuspended in 25-fold distilled water, sterilized in a high-pressure steam sterilizer (121 °C, 20 min, 0.1 MPa), and cooled to ambient temperature. A 10% (*w*/*v*) inoculum of *Lactobacillus plantarum P9*: *Lactobacillus rhamnosus* (3:1) was introduced into the medium, followed by fermentation at 37 °C in a shaking water bath. Samples were collected on days 0, 1, 2, 3, 4, and 5 of fermentation, and centrifuged immediately to obtain the supernatant for analysis.

The fermented supernatant was concentrated to the same volume using a rotary evaporator at 40 °C under vacuum. The concentrated sample was prefrozen at −20 °C for 24 h and lyophilized for 48 h at −50 °C with a vacuum pressure of 10~15 Pa. The resulting lyophilized powder was sieved through a No. 3 Pharmacopoeia sieve for further analysis.

### 2.3. Physicochemical Properties Analysis

#### 2.3.1. Microbial Viability Determination

The colony-forming units (CFU) of *Lactobacillus* during the fermentation of *PL* were determined using the plate count method. Serial dilutions of the *PL* fermentation broth were prepared at multiples of 10^1^, 10^2^, 10^3^, 10^4^, 10^5^, 10^6^, and 10^7^. An appropriate dilution was inoculated onto De Man, Rogosa and Sharp (MRS) agar medium, and the plates were incubated at 37 °C for 48 h. Colony counting was performed in accordance with GB/T 4789.35-2008 [13], where plates with colony counts between 30 and 300 CFU were selected for enumeration. The final results were expressed as CFU·mL^−1^, adhering to the reporting standards for microbial enumeration in food science.

#### 2.3.2. Determination of pH and Titratable Acidity

The pH of fermented *PL* at different fermentation times was determined in accordance with GB 5009.237-2016 [14]. The total acid content was measured using the potentiometric titration method with a pH meter as specified in Section 2 of GB 12456-2021 [15].

#### 2.3.3. Quantification of Total Flavonoids, Total Phenolics, and Reducing Sugars

The total flavonoids content in the *PL* fermentation broth was determined using an ultraviolet spectrophotometric method [16]. Briefly, the sample from Section 2.2 was diluted to an appropriate concentration with 30% ethanol and zeroed with the same solvent. The absorbance was measured at 250 nm. The content was calculated based on a calibration curve prepared using puerarin and expressed as micrograms of puerarin equivalents per milliliter (μg·mL^−1^).

The total phenolic content was determined using the Folin–Ciocalteu method [17]. Briefly, 20 μL of appropriately water-diluted sample from Section 2.2 was transferred to a 96-well microplate, followed by the addition of 80 μL of 0.1 mol·L^−1^ Folin–Ciocalteu reagent. The mixture was incubated in the dark for 5 min. Then, 100 μL of 7.5% sodium carbonate solution was added and the reaction was allowed to proceed at room temperature for 30 min. The absorbance was measured at 765 nm. The content was calculated based on a calibration curve prepared using gallic acid and expressed as milligrams of gallic acid equivalents per milliliter (mg·mL^−1^).

The reducing sugar content was determined using the DNS method [18]. Briefly, 0.20 mL sample from Section 2.2 was transferred to test tubes, diluted with water to 1.00 mL, and then supplemented with 2.00 mL of DNS reagent. The mixture was heated in a boiling water bath for 5 min. After immediate cooling, 9.00 mL of water was added. The absorbance was measured at 540 nm after thorough mixing. The content was calculated based on a calibration curve prepared using glucose and expressed as milligrams of glucose equivalents per milliliter (mg·mL^−1^).

#### 2.3.4. Antioxidant Capacity Assessment

The DPPH, ABTS^+^, OH radical scavenging activity were determined based on the method of [19], with slight modifications. The *PL* fermentation broth from Section 2.2 was diluted with 30% ethanol. Then, 25 μL of the diluted sample was mixed with 175 μL of 0.10 mmol·L^−1^ DPPH-ethanol, incubated at room temperature in darkness for 60 min, and the absorbance was measured at 517 nm (A_1_). A blank control was prepared by replacing the DPPH solution with absolute ethanol (A_2_), while the sample was replaced with ethanol for the reference (A_0_). The DPPH Scavenging Activity (%) = $\left(1-\frac{A_{1}-A2}{A_{0}}\right)\times 100\%$. The results were expressed as μmol Trolox equivalent (TE) per gram of *PL* decoction pieces (μmol TE·g^−1^ *PL*), using Trolox as the antioxidant reference standard.

The *PL* fermentation broth from Section 2.2 was diluted with water. Then, 25 μL of the diluted sample was mixed with 175 μL of ABTS^+^ solution, incubated at room temperature in darkness for 40 min, and the absorbance was measured at 734 nm (A_1_). The absorbance of the diluted ABTS^+^ solution without the sample was recorded as A_0_. The ABTS^+^ Scavenging Activity (%) = $\frac{A_{0}-A_{1}}{A_{0}}\times 100\%$. The results were expressed as μmol Trolox equivalent (TE) per gram of *PL* decoction pieces (μmol TE·g^−1^ *PL*), using Trolox as the antioxidant reference standard.

1 mL salicylic acid solution was added to a 10 mL test tube, followed by the addition of 1 mL FeSO_4_ solution, 1 mL of appropriately water-diluted sample from Section 2.2, and 1 mL H_2_O_2_ solution. The mixture was incubated at 37 °C in a water bath for 30 min, and the absorbance was measured at 510 nm, denoted as A_1_. A blank sample was prepared by replacing the 1 mL sample with 1 mL distilled water, and its absorbance was recorded as A_0_. The ·OH Scavenging Activity (%) = $\frac{A_{0}-A_{1}}{A_{0}}\times 100\%$. The results were expressed as μmol Vitamin C (VC) per gram of *PL* decoction pieces (μmol VC·g^−1^ *PL*), using Vitamin C as the antioxidant reference standard.

The ferric reducing antioxidant power (FRAP) assay was performed based on the method of [20], with slight modifications. A 20 μL of appropriately water-diluted sample from Section 2.2 was added to 200 μL FRAP reagent, incubated at room temperature in darkness for 30 min, and the absorbance was measured at 593 nm (A_1_). For the reference, FeSO_4_ solution was used instead of the sample (A_0_), while water was used as the blank (A_2_). The ferric reducing ability was expressed as FeSO_4_ equivalents (μmol FeSO_4_·g^−1^ *PL*).

### 2.4. Flavor Profiling of PL Fermentation via Intelligent Sensory Technology

#### 2.4.1. Flavor Discrimination Using Electronic Tongue and Electronic Nose Technologies

The 30 mL of fermented *PL* samples taken from different fermentation times were measured accurately as described in Section 2.2, set the sampling time to 120 s, and sensors such as AAE, CT0, CA0, C00, AE1, and GL1 were used to detect umami, saltiness, acidity, bitterness, astringency, and sweetness, respectively [21]. Accurately weighed 1.0 g samples of freeze-dried extract powder of *PL* from different fermentation times as described in Section 2.2 were placed in the corresponding sample vials of the electronic nose instrument. The samples were allowed to stand at room temperature for 30 min to achieve headspace saturation with sample gas. The instrument cleaning time was set to 180 s, the gas flow rate to 0.6 L·min^−1^, the detection time to 120 s, and the sample volume to 10 mL, with three replicates per group. The results were visualized using radar charts.

#### 2.4.2. Analysis via Heracles NEO Ultra-Fast Gas Chromatography Electronic Nose

A 1.00 g sample powder from Section 2.2 was accurately weighed and transferred to a headspace vial, and subsequently analyzed according to the specified detection conditions [22]. The automatic injection conditions were set as follows: incubation time of 15 min, incubation temperature of 70 °C, agitation for 1 s, no agitation for 20 s, washing time of 90 s, syringe temperature of 60 °C, and filling speed of 500 μL·s^−1^. The instrument conditions included an injection volume of 3000 μL, injection flow rate of 125 μL·s^−1^, injector temperature of 200 °C, injector pressure of 10 kPa, trap collection temperature of 40 °C, and initial column oven temperature of 50 °C. The temperature program was set to increase from 50 °C to 80 °C at a rate of 1 °C·min^−1^, then further increase to 250 °C at 3 °C·min^−1^, followed by a 21 s hold. The detection time was 110 s, with a data acquisition period of 0.01 s. Analysis was performed using the MXT-1701 polar capillary column and the MXT-5 weakly polar capillary column (Alpha MOS, Toulouse, France).

### 2.5. Analysis of Non-Volatile Components

Unfermented/fermented *PL* liquid from Section 2.2 was mixed with methanol (1:4) and ultrasonicated at 4 °C for 30 min. The mixture was centrifuged at 14,600× *g* and 4 °C for 15 min, and the supernatant was collected. The supernatant was then mixed with three fold volume of methanol, centrifuged at 16,760× *g* and 4 °C for 10 min, and the supernatant was collected. The supernatant was evaporated using a highspeed vacuum concentrator, redissolved in 100 μL of methanol-water (1:1, *v*/*v*), and centrifuged at 16,760× *g* and 4 °C for 15 min. The supernatant was used for subsequent analysis.

The chromatographic analysis was performed on an ACQUITY UPLC HSS T3 column (2.1 mm × 100 mm, 1.8 μm; Waters, Milford, MA, USA) with a mobile phase of 0.1% formic acid in water (A) and acetonitrile (B). The gradient elution was as follows: 0–35 min, 5–25% B; 35–40 min, 25–95% B; 40–42 min, 95% B; 42–45 min, 95–5% B. Chromatographic separation was achieved on a Shimadzu LC-30AD system with an injection volume of 1.0 μL, flow rate of 0.25 mL/min, column temperature of 40 °C, and a 45 min gradient. Data were acquired using Data-Dependent Acquisition (DDA) mode, with positive and negative ion modes analyzed in separate runs. Key parameters included: spray voltage of +5500 V (positive) and −4500 V (negative); declustering potential of +100 V (positive) and −100 V (negative); collision energy of 35 ± 15 eV (positive) and −35 ± 15 eV (negative); curtain gas of 40.0 (positive) and 40.0 (MS1)/35.0 (MS2) (negative). Common parameters included: ion source temperature of 500 °C; nebulizer gas (GS1) of 50.0; dryer gas (GS2) of 50.0. Full MS scans (*m*/*z* 100–1500) were acquired with accumulation time of 250 ms; MS/MS scans (*m*/*z* 50–1250) were triggered at intensity >10 counts using rolling collision energy with accumulation time of 100 ms. Maximum of 6 MS/MS spectra per 900 ms cycle were collected over 45 min [23].

Chemical components of *PL* were identified using databases such as PubChem (https://pubchem.ncbi.nlm.nih.gov/, accessed on 20 May 2025), MassBank (https://massbank.eu/MassBank/, accessed on 20 May 2025), and literature databases such as China National Knowledge Infrastructure (CNKI), PubMed, and SciFinder. A chemical database was established for component identification.

### 2.6. Network Pharmacological Analysis

#### 2.6.1. Screening of Active Components and Prediction of Their Putative Targets

Based on the newly generated components and key significantly upregulated components of fermented *PL* as candidate active ingredients, their chemical structures were retrieved from the PubChem database (https://pubchem.ncbi.nlm.nih.gov/, accessed on 20 June 2025). These structures were subsequently uploaded to the SwissADME online platform (http://swissadme.ch/, accessed on 20 June 2025) to screen for pharmacokinetic parameters relevant to treating hyperlipidemia. The selected active components were then used to obtain their 2D structures and SMILES encodings from the PubChem database. The 2D structures were submitted to the SwissTargetPrediction database (http://www.swisstargetprediction.ch/, accessed on 20 June 2025) to predict their corresponding protein targets based on structural similarity.

#### 2.6.2. Collection of Disease-Associated Targets and Screening of Intersection Targets

Using “Hyperlipidemia” as the keyword, searches were conducted in the OMIM database (https://www.omim.org/, accessed on 20 June 2025), DrugBank database (https://go.drugbank.com/, accessed on 20 June 2025), GeneCards disease database (https://www.genecards.org/, accessed on 20 June 2025), and DisGeNET database (https://www.disgenet.org/, accessed on 20 June 2025) to identify disease-related targets. The following evidence-score thresholds were applied: In GeneCards, only targets with a Relevance score greater than 0.6 were retained; In DisGeNET, a GDA Score threshold of ≥0.4 was set; in DrugBank, only targets of approved drugs were included; no specific score filter was applied to OMIM due to its curated nature. The resulting target sets from each database were merged and deduplicated based on official gene symbols (HGNC) to create a comprehensive yet high-confidence hyperlipidemia target library. Subsequently, the active ingredient targets and disease targets were imported into the Bioinformatics software (http://www.bioinformatics.com.cn/, accessed on 20 June 2025) to identify common targets, and a Venn diagram was generated using the Venny 2.1 tool (https://bioinfogp.cnb.csic.es/tools/venny/, accessed on 20 June 2025) to visualize the intersection. Finally, the Cytoscape 3.8.2 (http://www.cytoscape.org/, accessed on 25 June 2025) was employed to construct a “bioactive compound-target-disease” network.

#### 2.6.3. Protein–Protein Interaction (PPI) Network Construction and Hub Target Screening

The PPI network was constructed by importing the intersection targets from Section 2.6.2 into the STRING database (https://string-db.org/, accessed on 20 June 2025) and setting the species to “*Homo sapiens*”. The interaction threshold was set to “medium confidence” (>0.4) to retrieve protein interaction data, which was then saved as a TSV file. This file was imported into Cytoscape 3.8.2 software for visualization of the PPI network. The CytoNCA plugin was employed to calculate six topological parameters: betweenness, closeness, degree, eigenvector, local average connectivity, and network centrality. Targets with values exceeding the median of these parameters were screened twice to identify core targets, ensuring robustness of the selection. The final network was visualized in Cytoscape 3.8.2 for further analysis.

#### 2.6.4. Gene Ontology (GO) and Kyoto Encyclopedia of Genes and Genomes (KEGG) Pathway Enrichment Analysis with Construction of the “Active Components-Targets-Pathways-Disease” Network

The intersection targets were imported into the DAVID database (https://davidbioinformatics.nih.gov/, accessed on 20 June 2025) for GO functional and KEGG pathway enrichment analysis, with *Homo sapiens* selected as the species. The GO functional analysis included Biological Process (BP), Molecular Function (MF), and Cellular Component (CC) categories. The *p*-values from the hypergeometric test were adjusted for multiple comparisons using the Benjamini–Hochberg false discovery rate (FDR) correction. An adjusted *p*-value (FDR) < 0.05 was considered statistically significant. The enrichment results were visualized using the Bioinformatics software (http://www.bioinformatics.com.cn/, accessed on 20 June 2025). The active ingredients, their interaction targets, and the top 20 KEGG pathways along with their associated genes were imported into Cytoscape 3.8.2 software to construct a “bioactive ingredients-target-pathway-disease” network, which integrates the molecular mechanisms of *PL* fermentation products in lipid-lowering effects.

### 2.7. Molecular Docking (MD) of Active Components with Hub Targets

The bioactive ingredients with higher degree values obtained from Section 2.6.1 were selected as ligands, while the core target proteins identified in Section 2.6.3 served as receptors for MD studies. The crystal structures of the core target proteins were retrieved from the Protein Data Bank (PDB) database (https://www.rcsb.org/, data release 1 January 2025, accessed on 28 June 2025) using the following accession identifiers: 4EJN (AKT1), 3POZ (EGFR), 2OVZ (MMP9), 1O8A (ACE), 8SBT (HSP90AA1), 8U8X (ERBB2), 6LXA (PPARA), 7AQF (SERPINE1), 3DZY (PPARG), and 1 × 7R (ESR1). The two-dimensional structures (2D) of small-molecule ligands were sourced from the PubChem database (https://pubchem.ncbi.nlm.nih.gov/, accessed on 28 June 2025). The following compounds were retrieved using their PubChem Compound ID (CID): genistein (5280961), formononetin (5280378), daidzein (5281708), glycitein (5317750), calycosin (5280448), biochanin A (5280373), pinocembrin 7-acetate (73554040), and luteolin (5280445). These 2D structures were then converted into three-dimensional (3D) formats and energy-minimized using Chem3D 20.0. The MD procedure was performed according to the methodology described in reference [24]. The results were calculated and output using AutoDock Tools 1.56, and further analyzed and visualized using PyMol 2.5.6.

### 2.8. Molecular Dynamics Simulation (MDS) of Active Components with Hub Targets

MDS of protein-ligand complex were performed to explore the interaction between the receptors and ligands by using GROMACS 2020.3 software [25]. The amber 99sb-ildn force field and the general Amber force field (GAFF) were used to generate the parameter and topology of proteins and ligands, respectively. Setting the size of the simulation box so that the distance between each atom of the protein and the box was greater than 1.0 nm. Filling the box with an explicit solvent-simple point charge model (SPC216 water molecules) and replacing the water molecules with Na^+^ and Cl^−^ counterions to make the simulation system electrically neutral. The entire system was optimized by the steepest descent method, so that the unreasonable contact or atom overlap in the system is reduced. To achieve sufficient pre-equilibration of the simulation system, the NVT ensemble and the NPT ensemble were performed for 100 ps at 300 K and 1 bar, respectively. Subsequently, the MDS of 100 ns was performed with periodic boundary conditions, and the temperature (300 K) and pressure (1 bar) were controlled by the V-rescale and Parrinello-Rahman methods, respectively [26]. The Newton equation of motion was calculated using the leapfrog integration with the time step of 2 fs. The long-range electrostatic interaction was calculated by the Particle Mesh-Ewald (PME) method using Fourier spacing of 0.16 nm, and the LINCS method was used to constrain all bond lengths. The binding free energy of the compound was calculated by gmx_mmpbsa.

### 2.9. Statistical Analysis

Data are presented as the mean ± standard deviation (SD) of triplicate experiments. Statistical analysis was performed using SPSS 26.0 software. Differences among groups were assessed by one-way analysis of variance (ANOVA) followed by Duncan’s post hoc test, with a *p*-value < 0.05 considered statistically significant. Data visualization, including radar charts and volcano plots, was conducted using Origin 2021 Pro. For metabolomic data analysis, Simca 14.1 software was used. Differential metabolites were identified based on a *p*-value < 0.05, a fold change (FC) ≥1.5 or ≤2/3, and a variable importance in projection (VIP) value >1. Analyses involving network pharmacology, molecular docking (MD), and molecular dynamics simulations (MDS) were performed using Cytoscape 3.8.2, AutoDock Tools 1.5.6, PyMol 2.5.6, and Gromacs-2020.3, respectively.

## 3. Results

### 3.1. Microbial Viability and Physicochemical Dynamics During PL Fermentation

*PL* is rich in various nutrients, such as starch and polysaccharides, which can serve as primary carbon and nitrogen sources for *Lactobacillus* [27]. As shown in Table 1, the *Lactobacillus* strains adapted well to the *PL* substrate. Bacterial growth was rapid during the initial fermentation phase, increasing from 7.23 to 8.89 lg CFU·mL^−1^ on the first day, after which the population stabilized. Concurrently, the pH of the broth decreased gradually over time, while the total acid content increased and stabilized by day 2. These changes are likely attributable to the production of organic acids, such as lactic acid and malic acid, by *Lactobacillus* [28].

The contents of total flavonoids, total phenols, and reducing sugars in the samples were calculated based on the following calibration curves: puerarin (A = 0.0721C + 0.017, R^2^ = 0.9992), gallic acid (A = 4.4502C + 0.0265, R^2^ = 0.9996), and glucose (A = 0.9768C − 0.0664, R^2^ = 0.9991), with the results shown in Table 1. The consumption of reducing sugars, such as glucose and fructose, decreased due to consumption by *Lactobacillus*. The total flavonoid content exhibited a stage-specific metabolic pattern: during the initial fermentation phase, *Lactobacillus* secreted cellulases and pectinases to degrade the cell wall of *PL*, which promoted the release of flavonoids and led to a significant increase in total flavonoid content from 124.73 to 129.76 mg g^−1^ [29]. In the later stages, the degradation of glycosidic bonds mediated by β-glucosidases resulted in a noticeable decrease in total flavonoid content, which declined to 113.91 mg g^−1^, representing a reduction of 8.67% compared to the pre-fermentation level. Overall, the flavonoid content was largely maintained. Total phenols fluctuate due to the catalytic action of polyphenol oxidases, which was closely related to the enzymatic molecular rearrangement mechanisms reported by An [19] and Zhou [30].

### 3.2. Dynamic Changes in Antioxidant Activity

The evaluation of antioxidant activity quantifies the ability of substances to scavenge free radicals and inhibit oxidative chain reactions, which is essential for screening natural antioxidants and assessing their potential health benefits [31]. As shown in Table 1, after 5 days of fermentation, the antioxidant capacity of *PL* broth showed varying degrees of change compared to its pre-fermentation state. The FRAP value decreased from 438.95 to 427.08 μmol FeSO_4_·g^−1^ *PL* (a 2.70% reduction). The DPPH radical scavenging activity declined from 113.71 to 95.13 μmol TE·g^−1^ *PL* (a 16.36% decrease). A more pronounced reduction was observed in ABTS^+^ radical scavenging activity, which dropped from 736.29 to 550.08 μmol TE·g^−1^ *PL* (a 25.29% decrease). The most significant change was noted in hydroxyl radical (OH) scavenging ability, which fell markedly from 89.55 to 37.96 μmol VC·g^−1^ *PL* (a 57.61% decrease).

Overall, although a statistically significant decrease was observed in the FRAP, DPPH, and ABTS^+^ radical scavenging abilities after fermentation, the activities were retained with more than 74.71% of the original capacity preserved. However, the·OH scavenging ability decreased sharply within the first day of fermentation. This rapid decline might be attributed to the biotransformation of native antioxidant components in *PL* that are particularly effective against highly reactive hydroxyl radicals into other compounds with different activity profiles. Thereafter, the scavenging activity stabilized, possibly compensated by the formation of microbial metabolites or transformed components in the later stages of fermentation [32].

### 3.3. Flavor Dynamics Characterization Using Intelligent Sensory Technologies

#### 3.3.1. Electronic Tongue and Electronic Nose Characterisation

**Figure 1:**
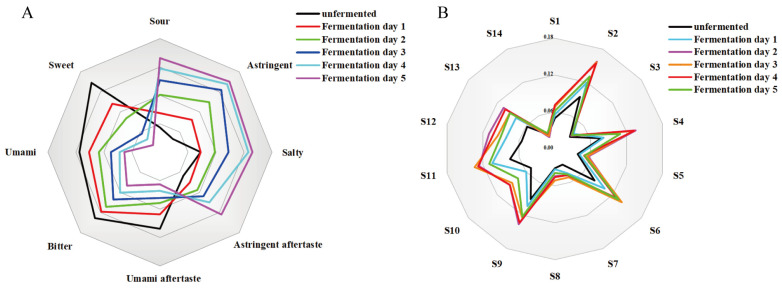
Radar chart of electronic tongue and electronic nose analyses for *PL* samples at different fermentation time points. (**A**) Electronic tongue measurement results of *PL* fermentation broth samples, (**B**) Electronic nose measurement results of *PL* fermentation broth samples.

Microbial fermentation significantly modifies the flavor profile of substrates. Intelligent sensory technologies, such as the electronic nose (E-nose) and electronic tongue (E-tongue), allow for the quantitative assessment of these changes by simulating human sensory perception [33]. These tools were employed to objectively evaluate the dynamic flavor changes during *PL* fermentation. As shown in Figure 1, the E-tongue analysis revealed distinct alterations in taste attributes: sourness, astringency, saltiness, and lingering astringency increased continuously, whereas umami, bitterness, sweetness, and lingering umami decreased throughout the process. Notably, although sourness increased from the early stages, its intensity surpassed the detection threshold only in the later phase of fermentation, indicating a significant shift in mouthfeel. Electronic nose sensor signals revealed a biphasic trend in odor intensity, with significant increased in signals corresponding to organic acid esters (S4), terpenes (S6), volatile organic compounds (S11), and decreased in signals for lactones (S14), sulfides (S12), and amines (S8), the features of sensors were shown in Table 2. These results suggest that *Lactobacillus* fermentation enriched the broth with floral and fruity ester-terpene compounds while suppressing sulfur-containing off-odors, thereby improving the overall aroma profile of *PL*.

#### 3.3.2. Heracles NEO Ultra Rapid Gas Phase Electronic Nose

Volatile odor compounds were qualitatively analyzed using both MXT-5 (non-polar) and MXT-1701 (moderately polar) chromatographic columns. Identification criteria required a peak area greater than 500 and a match factor exceeding 70 against the AroChemBase database [34].

As shown in Table 3, 22 potential odor compounds were detected in *PL* samples throughout fermentation. Only two compounds were identified prior to fermentation. The number of odor compounds increased significantly in the initial stages, reaching a maximum of 14 on day 2. During this period, the appearance or increased concentration of compounds such as β-pinene, 2-methyl nonane, (E)-2-Hexen-1-ol, 5-ethyl-3-hydroxy-4-methyl-2(5H)-furanone, and propylenglycol contributed to the evolving flavor profile. Specific compounds identified on day 3, including (E)-2-Hexen-1-ol butanoate (imparting apple, apricot, and banana notes), 5-ethyl-3-hydroxy-4-methyl-2(5H)-furanone (caramel and toffee-like), tridecane (orange-like), and L(-)-Carvone (basil and anise-like), constituted the characteristic fermented aroma. Although some flavors diminished on days 4 and 5, new compounds emerged, such as propylenglycol (alcoholic), 2,3-butanediol (creamy), and 5-methylfurfural (almond and coffee-like). In summary, co-fermentation with *Lactobacillus rhamnosus* and *L. plantarum P9* transformed *PL* components into diverse volatile flavors. While some compounds were degraded in later stages, others were generated or modified. This dynamic process enriched the profile with alcohols, alkanes, and esters, significantly enhancing the overall flavor of *PL* and providing a foundation for future product development [6].

### 3.4. Non-Volatile Component Dynamics

**Figure 2:**
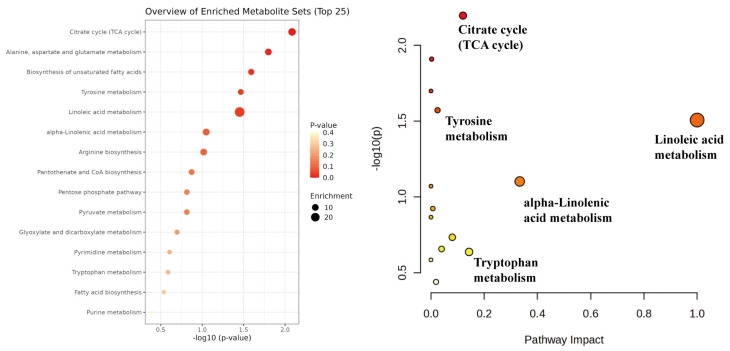
KEGG pathway enrichment analysis of differential metabolites. The larger dot represents a greater number of differentially expressed metabolites enriched in the pathway, while the darker color indicates a smaller *P*-value (indicating higher significance) in the enrichment analysis.

Non-targeted metabolomics analysis using UHPLC-Q-TOF/MS was performed to profile components in *PL* samples at different fermentation time points. A total of 182 common components were identified across all samples. Comparative analysis revealed that seven compounds, including 3-hydroxy-2-pyrone and uridine, disappeared during fermentation, whereas seven others, such as D-ribose and kaempferol, were newly generated. These changes are potentially associated with metabolic pathways including alanine, aspartate, and glutamate metabolism, arginine biosynthesis, and the pentose phosphate pathway (Figure 2).

**Figure 3:**
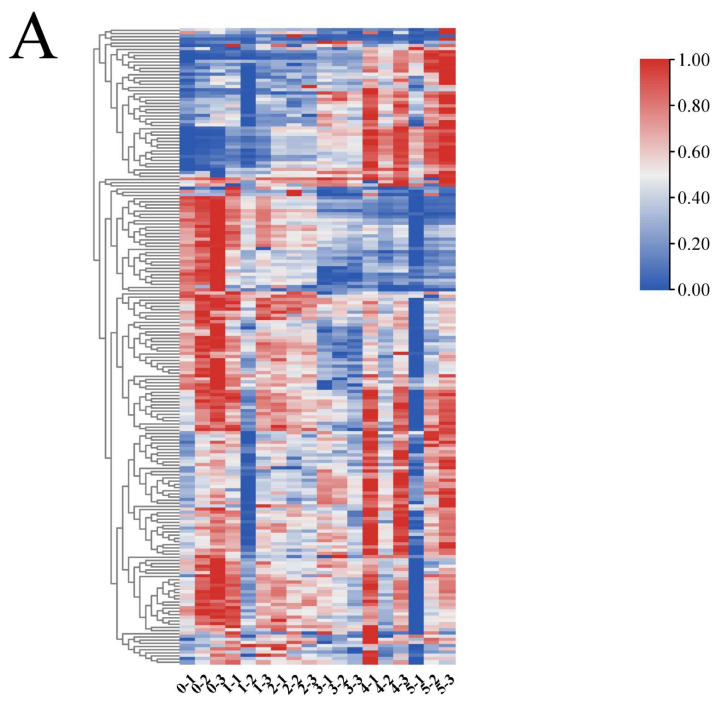

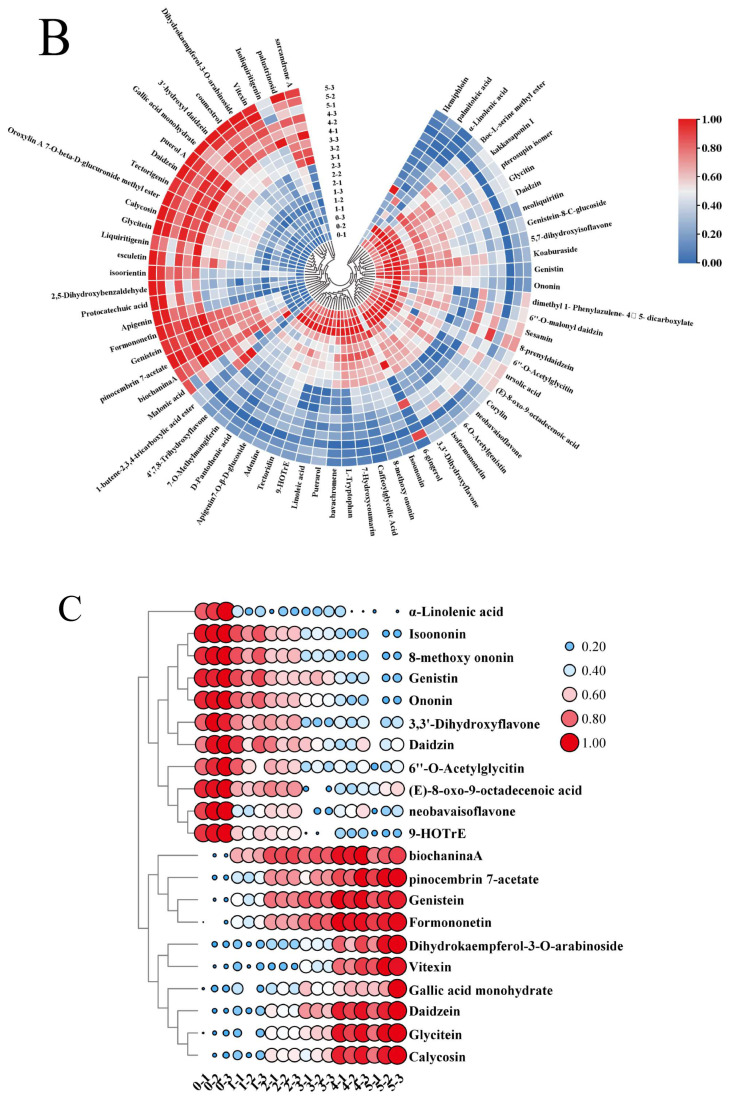
The clustering heatmap of differential active components during *PL* fermentation shows that the color gradient from blue to red represents a gradual increase in relative content. (**A**) Hierarchical clustering heatmap of relative abundance of common components in *PL* at different fermentation times, (**B**) Hierarchical clustering heatmap of significantly different components during *PL* fermentation, (**C**) Hierarchical clustering heatmap of key significantly different components in *PL* fermentation.

To visualize the dynamic changes in common components, hierarchical cluster analysis of 182 common features was performed and presented as a heatmap (Figure 3A). The results showed a clear clustering pattern, distinguishing components that increased from those that decreased in abundance during fermentation. Principal component analysis (PCA) of the peak area data further confirmed a distinct separation between fermented and unfermented samples within a 95% confidence interval. The model parameters (R^2^X > 0.5, Q^2^ > 0.5) indicated significant fermentation-induced alterations in the non-volatile metabolome [35].

**Figure 4:**
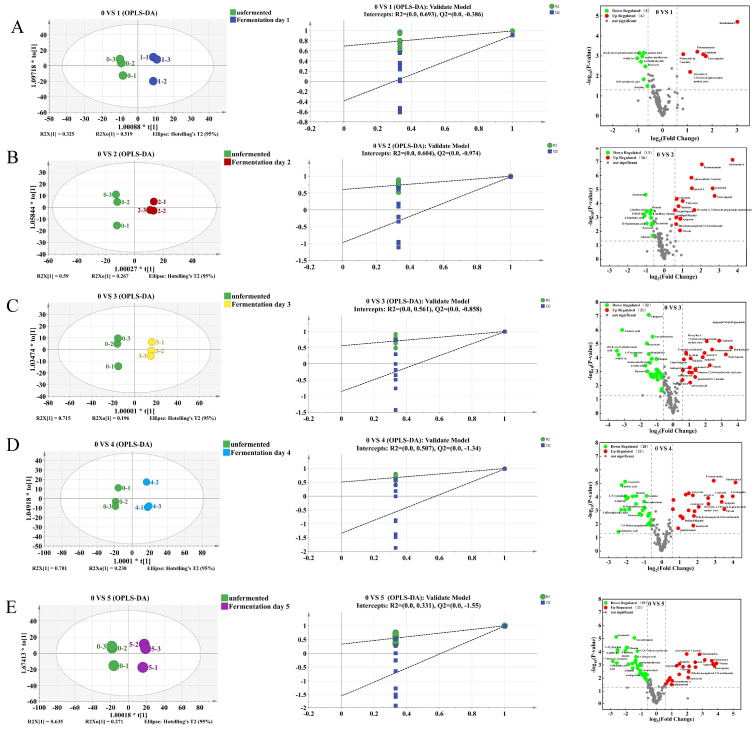
The OPLS-DA score plots, permutation test plots, and volcano plots of differential components between fermented and unfermented *PL* at days 1, 2, 3, 4, and 5. (**A**) Day 0 vs. Day 1, (**B**) Day 0 vs. Day 2, (**C**) Day 0 vs. Day 3, (**D**) Day 0 vs. Day 4, (**E**) Day 0 vs. Day 5. In the volcano plot, red dots represent components with an FC ≥ 1.5 and *p* < 0.05, green dots represent components with an FC ≤ 2/3 and *p* < 0.05, and gray dots represent components with 2/3 < FC < 1.5 or *p* > 0.05.

To enhance the separation and identify key biomarkers, orthogonal partial least squares-discriminant analysis (OPLS-DA) was employed. The models for comparing unfermented samples with each fermentation time point showed high explanatory (R^2^X, R^2^Y > 0.8) and predictive (Q^2^ > 0.9) power (Table 4, Figure 4). A permutation test (*n* = 200) demonstrated the models’ robustness without overfitting, as indicated by the negative Q^2^ regression line intercept [36].

Differential components were screened from the OPLS-DA models based on a fold change (FC) ≥1.5 or ≤2/3 and a *p*-value < 0.05, and visualized in a volcano plot (Figure 4). A total of 68 significantly differential active components were identified between fermented and unfermented *PL*. Specifically, on Day 1, 14 differential components were identified (6 upregulated, 8 downregulated). Notably, the aglycones genistein, formononetin, irisolidone, and glycitein were significantly upregulated, likely due to the β-glucosidase activity of *Lactobacillus* hydrolyzing glycosidic bonds [37]. Conversely, D-pantothenic acid, adenine, linoleic acid, and α-linolenic acid were downregulated, suggesting their biotransformation may via purine degradation and fatty acid metabolism pathways.

On day 2, 29 differential components were observed (16 upregulated, 13 downregulated). Upregulated components included apigenin, liquiritigenin, coumestrol, and calycosin, whereas apigenin-7-O-β-glucoside, tectoridin, ononin, and isononin were downregulated. Studies indicate that apigenin enhances fat oxidation and autophagy-mitochondrial pathways, accelerating lipid droplet degradation [38]. Liquiritigenin inhibits the PI3K-AKT signaling pathway, reducing the activity and expression of pro-matrix metalloproteinase-2 (proMMP-2), thereby inhibiting the migration of human lung adenocarcinoma A549 cells [39]. Coumestrol reduces liver malondialdehyde (MDA) levels and alanine aminotransferase/aspartate aminotransferase (ALT/AST) activity, increasing superoxide dismutase (SOD) activity to alleviate oxidative stress and inhibit hepatic fibrosis by regulating matrix metalloproteinase-1/tissue inhibitor of metalloproteinases-1 MMP-1/TIMP-1 balance and activating the janus kinase 2-Signal transducer and activator of transcription 3 (JAK2-STAT3) pathway [40].

On day 3, 53 differential components were identified, with 21 upregulated and 32 downregulated, palustrinosid and 3′-hydroxyl daidzein were significantly upregulated, while puerarin-7-O-xyloside, ursolic acid, and 6-gingerol were downregulated. On day 4, 50 differential components were detected, with 22 upregulated and 28 downregulated, gallic acid, esculetin, and isovitexin were upregulated, while sophoradioside and palmitic acid were downregulated. On day 5, 57 differential components were observed, with 22 upregulated and 35 downregulated, protocatechuic acid and 2,5-dihydroxybenzaldehyde were upregulated, while genistein-8-C-glucoside, glycitein, and daidzin were downregulated. Studies indicate that gallic acid can upregulate interferon regulatory factor 6 (IRF6) expression, negatively regulate the PPARγ signaling pathway, inhibit hepatic lipid accumulation, and alleviate liver injury [41]. Esculetin can activate the transcription factor CCAAT/enhancer-binding protein β (C/EBPβ), enhance the phagocytic activity of CD36 receptors on adipose tissue macrophages (ATMs), promote lipid clearance, increase HDL-C generation, and facilitate cholesterol excretion via bile acid-mediated reverse cholesterol transport [42]. Isoorientin can activate AMP-activated protein kinase (AMPK), upregulate PPARγ and uncoupling protein 1 (UCP1) expression, regulate the browning process of adipose tissue, and improve glucose uptake while inhibiting lipid accumulation [43].

The fermentation process induced substantial changes in the profile of active components in *PL*. By the mid-stage (day 3), *Lactobacillus* fermentation had significantly reshaped the metabolic landscape. Notably, it enhanced the content of components with established hypolipidemic activity, such as genistein, formononetin, and tectorigenin. These compounds are postulated to exert synergistic effects through multi-target and multi-pathway mechanisms, thereby reconstructing the material basis for the hypolipidemic efficacy of fermented *PL*. This multi-dimensional intervention in lipid metabolism disorders underscores the potential of fermented *PL* in regulating lipid metabolism, promoting adipose tissue health, and accelerating cholesterol clearance, providing a strong scientific basis for developing novel functional foods or adjunctive hypolipidemic agents.

To identify the core differential components, substances with a variable importance in projection (VIP) >1 were selected, yielding 21 key compounds. Cluster analysis visualized in a heatmap (Figure 3C) revealed two distinct clusters: 11 components (e.g., genistin, daidzin) decreased during fermentation, while 10 components (e.g., genistein, daidzein) increased. Given that aglycones like genistein and daidzein exhibit higher bioavailability and enhanced bioactivity, these 10 upregulated components were selected as enriched bioactive candidates for subsequent network pharmacology analysis targeting hyperlipidemia.

### 3.5. Network Pharmacological Analysis

#### 3.5.1. Screening of Key Enriched Active Components, Target Prediction, and Disease-Associated Target Acquisition

The SMILES representations of 17 newly generated and significantly upregulated components of fermented *PL*, including genistein, formononetin, gallic acid, were imported into the SwissADME online platform for analysis. The results identified genistein, formononetin, gallic acid, daidzein, glycitein, calycosin, biochanin A, pinocembrin 7-acetate, luteolin, and glycyl-L-isoleucine as key enriched active components of fermented *PL*. Their potential target proteins were predicted using the SwissTargetPrediction database, yielding 250 unique targets after deduplication.

**Figure 5:**
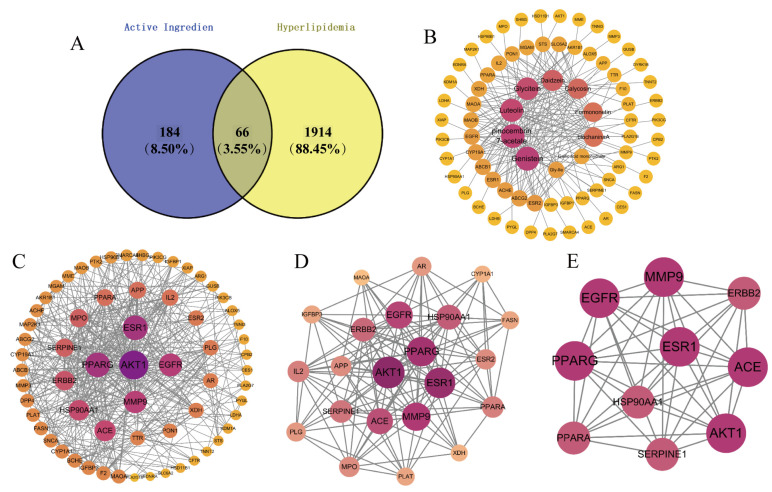
(**A**) Venn diagram of key enriched active components and hyperlipidemia targets, (**B**) Network diagram of ‘key enriched active components -hyperlipidaemia cross-targeting’, (**C**) Cross-targeted protein–protein interactions (PPI) network, (**D**) Core target protein interactions network after first screening, (**E**) Core target protein interactions network after second screening.

Disease-related targets were retrieved from the GeneCards, OMIM, DrugBank, and DisGeNET databases using the keyword “hyperlipidemia.” After integration and deduplication, 1980 hyperlipidemia-associated targets were collected. Intersection analysis between the component-related targets and disease targets identified 66 common targets (Figure 5A). A “component–target” network was constructed and visualized using Cytoscape 3.8.2 (Figure 5B). In this network, genistein, formononetin, and eight other differential active components exhibited high node sizes and color intensities, indicating a strong association with multiple target proteins. These components are likely core contributors to the lipid-lowering effects of *PL* fermentation, and further MD with their corresponding target proteins could provide critical insights into the mechanistic differences between fermented and unfermented *PL* in regulating hyperlipidemia.

#### 3.5.2. PPI Network Construction and Hub Target Identification

The 66 cross-target genes were imported into the STRING database to construct a PPI network using *Homo sapiens* as the species background. The interaction data were downloaded in TSV format and visualized using Cytoscape 3.8.2. In the resulting network, the size and color intensity of each node were proportional to its degree value. To identify core targets, the CytoNCA plugin was used for two rounds of topology-based screening [44]. The first screening round applied the following criteria: betweenness > 25.85, closeness > 0.52, degree > 12, eigenvector > 0.10, local average connectivity (LAC) > 6.42, and network score > 7.74, which yielded 22 targets and 140 edges (Figure 5D). The second screening applied stricter criteria: betweenness > 3.94, closeness > 0.69, degree > 11.5, eigenvector > 0.19, LAC > 8.17, and network score > 9.61, resulting in 10 core targets and 43 connections (Figure 5E), with detailed information presented in Table 5.

Literature evidence supports the roles of these core targets in relevant pathways. For instance, HSP90AA1 promotes tumor development by binding to STAT3. Luteolin, however, inhibits this interaction, induces phosphorylated STAT3 degradation via the proteasome pathway, and ultimately promotes tumor cell apoptosis [45]. Genistein selectively binds and activates PPARα and PPARγ receptors, reverses cell damage caused by high glucose, and protects endothelial cells [46]. Formononetin activates the farnesol X receptor (FXR), upregulates its target gene Shp expression, inhibits phosphoenolpyruvate carboxylase (Pepck), glucose-6-phosphate (G-6-pase), and sterol regulatory element-binding protein-1c (SREBP-1c) expression, reduces lipid synthesis, and enhances free fatty acid β-oxidation and insulin signaling pathway expression, thereby improving lipid metabolism disorders and insulin resistance [47]. These findings suggest that the key enriched active components in fermented *PL* may exert lipid-regulating effects by interacting with these core targets.

#### 3.5.3. Functional Enrichment of GO and KEGG Pathways with Construction of the “Component-Target-Pathway-Disease” Network

**Figure 6:**
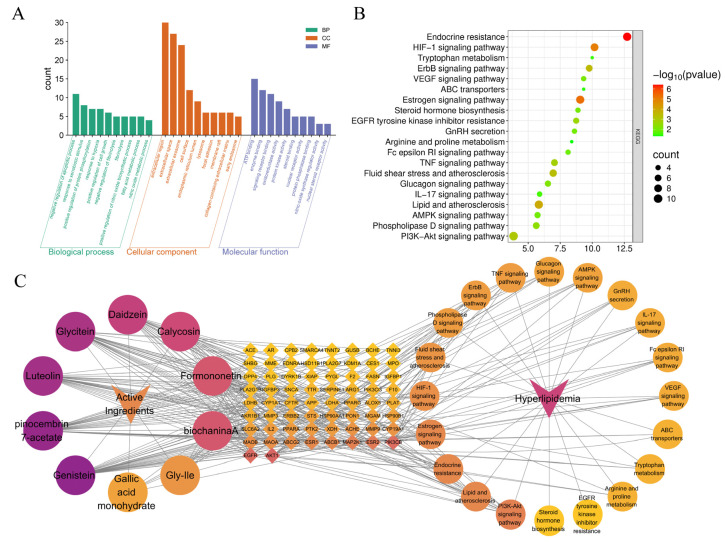
The GO and KEGG pathways of key enriched active components lipid-lowering effects. (**A**) The bar graph of the top 10 GO terms, including biological process (BP), cellular compound (CC), and molecular function (MF), (**B**) The bubble diagram of the top 20 KEGG pathways, (**C**) An integrated network of “components—targets—pathways—diseases”.

To further investigate the potential mechanisms through which the key enriched active components alleviate hyperlipidemia, GO functional and KEGG pathway enrichment analyses were performed on the 66 intersecting targets using the DAVID platform. The selected species was *Homo sapiens*, with a screening criterion of *p* < 0.04. The GO enrichment analysis ultimately identified 135 biological processes (BP), 40 cellular components (CC), and 69 molecular functions (MF). The top 10 related pathways were visualized, as shown in Figure 6A. The biological processes (BP) were primarily concentrated in signal transduction and apoptosis regulation (negative regulation of apoptotic process, positive regulation of protein phosphorylation); immune and inflammatory responses (positive regulation of nitric oxide biosynthetic process, response to xenobiotic stimulus, response to hypoxia); and lipid synthesis and metabolism (fatty acid metabolic process, nitric oxide metabolic process). Cellular components (CC) were mainly distributed in extracellular regions and matrix structures (extracellular region, extracellular space, collagen-containing extracellular matrix, extracellular exosome); cell membrane and adhesion microdomains (cell surface, membrane raft, focal adhesion); and intracellular organelles (endoplasmic reticulum lumen, lysosome, early endosome). Molecular functions (MF) were predominantly involved in protease activity regulation (protein kinase activity, nitric-oxide synthase regulator activity, protein phosphatase binding); signal transduction and receptor binding (signaling receptor binding, enzyme binding, nuclear steroid receptor activity); nuclear receptors and transcriptional regulation (ATP binding, steroid binding, nuclear receptor activity); and oxidative-reductase systems (oxidoreductase activity). Additionally, KEGG pathway analysis revealed that the intersecting targets primarily participated in signal transduction and disease-related pathways (endocrine resistance, HIF-1 signaling pathway, ErbB signaling pathway, TNF signaling pathway, AMPK signaling pathway, and 10 other metabolic pathways); metabolism and biosynthesis pathways (tryptophan metabolism, ABC transporters, estrogen signaling pathway, steroid hormone biosynthesis, and 7 other metabolic pathways); and cell apoptosis and cancer-related pathways (p53 signaling pathway, EGFR tyrosine kinase inhibitor resistance, lipid and atherosclerosis). It is noteworthy that the PI3K-AKT signaling pathway, fluid shear stress, and atherosclerosis are closely associated with hyperlipidemia [48,49]. The top 20 KEGG pathways, along with relevant target genes, active ingredients from Section 3.5.1, and intersecting targets, were imported into Cytoscape 3.8.2 software to construct a “component-target-pathway-disease” visualization network, as shown in Figure 6C. The results demonstrated that the key enriched active components exert lipid-lowering effects through multiple pathways, including the PI3K-AKT signaling pathway, fluid shear stress and atherosclerosis, endocrine resistance, estrogen signaling pathway, and others.

### 3.6. Molecular Docking

**Figure 7:**
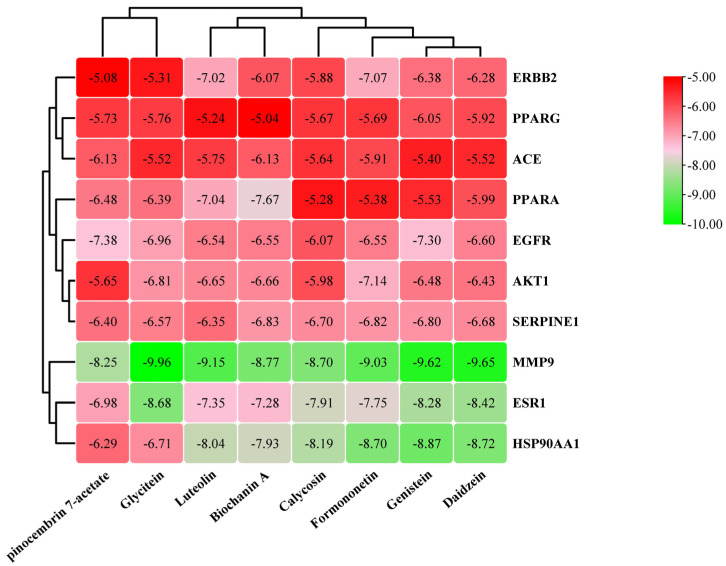
Hierarchically clustered heatmap of MD affinities between key enriched active components and hub targets. The color gradient of the grid squares, transitioning from green to red, represents a gradual increase in binding energy.

Molecular docking (MD) is a computational method widely used to predict the binding mode and affinity between small molecules and target proteins, which is essential for understanding drug-protein interactions [50]. In this study, MD simulations were conducted to evaluate the interactions between 8 core components (e.g., genistein, formononetin) and 10 core target proteins (e.g., EGFR, ESR1, PPARG, MMP9, HSP90AA1). The clustering analysis of binding energy distribution is shown in Figure 7. Generally, a docking binding energy lower than 0 kcal/mol indicates spontaneous binding, while values below −5 kcal/mol suggest strong binding affinity, and those below −7 kcal/mol indicate excellent binding activity [51]. Notably, all core components exhibited binding energies below –5.0 kcal/mol with the core targets, confirming robust binding affinity. In particular, MMP9, ESR1, and HSP90AA1 showed superior binding affinity relative to other targets, implying their potential as key mediators of the lipid-lowering effects of fermented *PL*. The generally consistent binding affinities among the core components suggest that isoflavone aglycones such as genistein and formononetin are primarily responsible for the differential lipid-lowering effects observed during fermentation.

**Figure 8:**
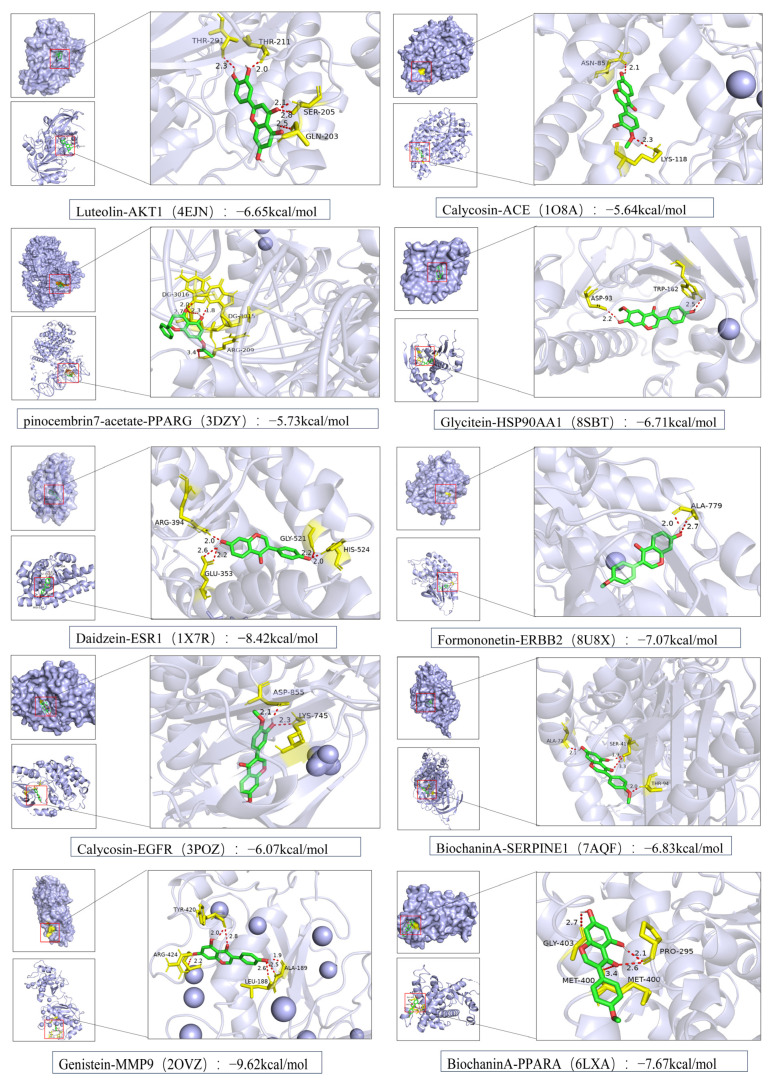
Key enriched active components and partial core target protein docking mode diagram. The left subfigure shows the binding pocket location between the compound and the protein. The right detailed view depicts the active component in green, amino acid residues in yellow, and hydrogen bonds represented by red dashed lines.

As shown in Figure 8, MD were performed using PyMOL 2.5.6 to analyze the interaction patterns between selected flavonoids and their corresponding target proteins. Daidzein formed hydrogen bonds with ARG-394, GLU-353, GLY-521, and HIS-524 residues of ESR1 (PDB ID: 1X7R); pinocembrin7-acetate established hydrogen bonds with DG-3016, DG-3015, and ARG-209 residues of PPARG (PDB ID: 3DZY); luteolin interacted with THR-291, THR-211, SER-205, and GLN-203 residues of AKT1 (PDB ID: 4EJN); calycosin bound to ASP-855 and LYS-745 residues of EGFR (PDB ID: 3POZ); genistein formed hydrogen bonds with TYR-420, ARG-424, ALA-189, and LEU-188 residues of MMP9 (PDB ID: 2OVZ); calycosin also interacted with ASN-85 and LYS-118 residues of ACE (PDB ID: 1O8A); glycitein established hydrogen bonds with ASP-93 and TRP-162 residues of HSP90AA1 (PDB ID: 8SBT); formononetin bound to ALA-779 residue of ERBB2 (PDB ID: 8U8X); biochaninA interacted with GLY-403, PRO-295, and MET-400 residues of PPARA (PDB ID: 6LXA); and biochanin A also formed hydrogen bonds with ALA-72, SER-41, and THR-94 residues of SERPINE1 (PDB ID: 7AQF). These results provide structural evidence that the key active components bind specifically to hyperlipidemia-associated targets, supporting the reliability of the network pharmacology predictions and highlighting the multi-target mechanism underlying the lipid-lowering activity of fermented *PL*.

### 3.7. Molecular Dynamics Simulation

**Figure 9:**
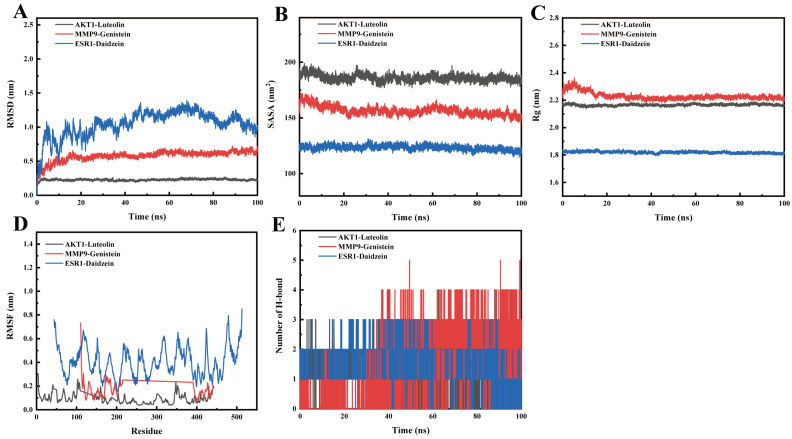
The time-dependent curves of Root Mean Square Deviation (RMSD) for the protein-ligand complexes of AKT1-Luteolin, MMP9-Genistein, and ESR1-Daidzein during the simulation process (**A**), the changes in Solvent Accessible Surface Area (SASA) over time (**B**), the variation in Radius of Gyration (Rg) over time (**C**), the Root Mean Square Fluctuation (RMSF) over time, (**D**) and the changes in hydrogen bonds over time are presented (**E**).

To evaluate the stability of the binding mode between active ingredients and key target proteins, we selected AKT1-Luteolin, MMP9-Genistein, and ESR1-Daidzein for MDS. As shown in Figure 9A, all three systems reached equilibrium within 10 ns, 10 ns, and 50 ns, respectively, with root-mean-square deviation (RMSD) values of 0.23 ± 0.01 nm, 0.58 ± 0.08 nm, and 1.04 ± 0.19 nm, indicating stable conformational binding and reliable simulation quality. The root-mean-square fluctuation (RMSF) values were 0.09 nm, 0.17 nm, and 0.41 nm, respectively, all below 0.8 nm, reflecting favorable structural adaptability under dynamic conditions (Figure 9D). Analysis of the radius of gyration (Rg) showed that the Rg values of the AKT1–Luteolin and ESR1–Daidzein complexes remained largely constant over time, suggesting no major change in structural compactness, while the MMP9–Genistein complex exhibited a decrease in Rg, implying enhanced compactness and stable ligand binding. These observations were further corroborated by solvent-accessible surface area (SASA) analysis (Figure 9B,C). Hydrogen bond analysis revealed average intermolecular hydrogen bond numbers of 1.08, 1.38, and 1.75 for the three complexes, respectively (Figure 9E), demonstrating consistent protein–ligand interaction. To quantitatively evaluate binding affinity, the binding free energies were computed using the MM/PBSA method via the gmx_mmpbsa script (https://jerkwin.github.io/gmxtools, accessed on 6 August 2025), which decomposes the total energy into electrostatic, van der Waals, and solvation contributions [52]. The total binding free energies for the AKT1–Luteolin, MMP9–Genistein, and ESR1–Daidzein complexes were –53.684 kJ/mol, –89.412 kJ/mol, and –78.322 kJ/mol, respectively (Table 6), indicating strong binding driven primarily by van der Waals and electrostatic interactions.

## 4. Discussion

*PL* is a traditional medicinal and edible resource valued for its pharmacological efficacy and nutritional value [2]. In our study, we used a co-culture of *Lactobacillus rhamnosus* and *L. plantarum P9* to ferment *PL*. We found that *Lactobacillus* fermentation significantly altered the flavor profile of *PL*, which may improve its suitability as an ingredient in functional beverages. Although electronic tongue and nose provide objective measurements, combining human sensory evaluation in future work remains essential for a comprehensive flavor assessment [53]. Due to technical limitations, the ultra-fast GC electronic nose used here is convenient and rapid but lacks sufficient accuracy. Subsequent studies should apply gas chromatography–mass spectrometry (HS-SPME–GC–MS) combined with electronic nose analysis to better characterize volatile compounds and interpret dynamic flavor changes during fermentation.

Another point of discussion involves the chromatographic separation of non-volatile compounds. Although the UPLC HSS T3 (C18) column effectively resolves flavonoids, it performs poorly for highly polar organic acids, amino acids, and highly hydrophobic aglycones, leading to incomplete metabolome coverage [54]. This limitation suggests that the rapid loss of hydroxyl radical scavenging activity on the first fermentation day should not be solely attributed to glycoside reduction; rather, the consumption of specific phenolic acids may contribute. The decrease in these acids also makes it difficult to directly link compositional changes to sensory profiles. In subsequent investigations, orthogonal chromatographic strategies—such as hydrophilic interaction liquid chromatography (HILIC)—will be adopted to extend the coverage of polar metabolites and enable a more comprehensive understanding of the transformation of bioactive components during *Lactobacillus* fermentation.

*PL* is rich in isoflavones, but these mainly exist as glycosides, which require microbial conversion to aglycones for absorption, limiting their bioavailability [55]. We demonstrated that *Lactobacillus* fermentation converts glycosylated isoflavones into aglycones (e.g., daidzein, genistein, formononetin) via specific microbial enzymes. The marked increase in these aglycones suggests improved absorption and hypolipidemic potential. Network pharmacology indicated that daidzein and other aglycones in fermented *PL* may bind to targets including AKT1, MMP9, and PPARA, potentially regulating lipid metabolism via PI3K-AKT, PPAR, and estrogen signaling pathways. Previous studies have reported that *PL* isoflavones activate PPARα and PPARγ, modulate lipid metabolism gene expression, and upregulate LDL receptor activity to enhance cholesterol clearance [56]. Formononetin also upregulates ATP-Binding Cassette Subfamily A/G Member 1 (ABCA1/ABCG1) and activates PPARγ/LXRα and PPARγ/HO-1 pathways to stimulate reverse cholesterol transport [57]. As phytoestrogens structurally similar to 17β-estradiol, daidzein and related compounds bind Estrogen Receptor α (ERα) and Estrogen Receptor β (ERβ), upregulate hepatic LDL receptor expression, promote LDL-C clearance, and induce Cytochrome P450 Family 7 Subfamily A Member 1 (CYP7A1) activity to facilitate cholesterol conversion and biliary excretion, collectively reducing serum cholesterol levels [58]. These findings support the hypothesis that fermented *PL* modulates lipid metabolism via PI3K–AKT, PPAR, and estrogen signaling pathways.

Nevertheless, we acknowledged that the network pharmacology approach employed here has inherent limitations. Databases such as STRING rely on curated protein–protein interactions from published literature, which biases predictions toward well-studied targets over under-characterized “dark targets.” Computational predictions only indicate potential binding interactions and carry a risk of false positives [59]. Moreover, database incompleteness and curation biases increase uncertainty in target and pathway predictions. Therefore, experimental validation is essential in future research. For example, studies using ovariectomized rat models could investigate whether fermented *PL* improves lipid profiles via estrogen-like effects, and cellular or molecular assays could examine whether it modulates lipid metabolism by activating PI3K–AKT or other signaling pathways, thereby providing more robust biological evidence for its hypolipidemic mechanisms.

## 5. Conclusions

This study systematically analyzed the comprehensive effects of *Lactobacillus* fermentation on the physicochemical properties, flavor profile, and transformation of active components in *PL*. The results indicated that the fermentation process largely preserved the total flavonoids, total phenolic content of *PL*, while significantly improving its flavor attributes and specifically promoting the enrichment of low-abundance isoflavone aglycones such as daidzein and genistein. Further, network pharmacology analysis combined with MD and MDS predicted that key enriched components in fermented *PL* could stably bind to core targets—including AKT1, MMP9, and PPARA—via hydrogen bonding and hydrophobic interactions, thereby regulating lipid metabolism homeostasis through multiple signaling pathways such as PI3K-AKT, PPAR, and estrogen signaling. In summary, this study established a multidimensional interaction network of “enriched components–core targets–signaling pathways–hyperlipidemia,” predicting the potential mechanisms and pathways underlying the hypolipidemic effects of fermented *PL*. These findings provide a theoretical basis for the development of aglycone-oriented functional products derived from *PL* for lipid management. Future studies should further validate the actual lipid-modulating effects and pathway mechanisms of multi-component synergies in fermented products using in vitro cell models and in vivo hyperlipidemic animal experiments.

## Figures and Tables

**Table 1:** Changes in microbial viability, physicochemical indicators, and antioxidant activity during the fermentation process of *PL*.

Bacterial Viability and Physical and Chemical Indicators	Fermentation Time					
	Day 0	Day 1	Day 2	Day 3	Day 4	Day 5
Bacterial Count/lgCFU·mL^−1^	7.23 ± 0.05 ^c^	8.89 ± 0.04 ^a^	8.97 ± 0.06 ^a^	8.98 ± 0.04 ^a^	8.89 ± 0.02 ^a^	8.74 ± 0.04 ^b^
Fermentation Solution pH	5.95 ± 0.05 ^a^	5.14 ± 0.06 ^b^	4.56 ± 0.07 ^c^	4.28 ± 0.03 ^d^	4.16 ± 0.03 ^de^	4.09 ± 0.03 ^e^
Total Acid Content/g·L^−1^	1.02 ± 0.03 ^f^	1.43 ± 0.02 ^e^	2.32 ± 0.05 ^d^	2.99 ± 0.02 ^c^	3.32 ± 0.03 ^b^	3.48 ± 0.03 ^a^
Total Flavonoid Content/mg·g^−1^	124.73 ± 1.24 ^b^	129.76± 1.15 ^a^	118.72 ± 1.50 ^c^	129.00 ± 1.68 ^a^	116.32 ± 0.68 ^cd^	113.91 ± 1.86 ^d^
Total Phenol Content/mg·g^−1^	48.81 ± 0.70 ^a^	45.77 ± 1.87 ^c^	47.72 ± 1.34 ^ab^	45.53 ± 1.27 ^c^	47.02 ± 0.97 ^bc^	48.39 ± 1.88 ^ab^
Reducing Sugar Content/mg·g^−1^	98.11 ± 1.71 ^a^	74.09 ± 0.78 ^b^	62.16 ± 0.87 ^c^	45.15 ± 0.87 ^e^	35.32 ± 0.99 ^f^	50.01 ± 1.53 ^d^
DPPH Radical Scavenging Capacity/μmol TE·g^−1^ *PL*	113.71 ± 3.68 ^a^	103.49 ± 1.91 ^b^	101.44 ± 3.95 ^b^	101.99 ± 3.03 ^b^	101.84 ± 2.29 ^b^	95.13 ± 4.66 ^c^
ABTS·+ Radical Scavenging Capacity/μmol TE·g^−1^ *PL*	736.29 ± 39.28 ^a^	663.86 ± 8.19 ^b^	599.51 ± 4.47 ^c^	642.39 ± 28.09 ^b^	585.19 ± 14.67 ^c^	550.08 ± 17.29 ^d^
OH·Radical Scavenging Capacity/μmol VC·g^−1^ *PL*	89.55 ± 1.04 ^a^	47.61 ± 1.10 ^b^	47.71 ± 2.52 ^b^	48.03 ± 1.31 ^b^	42.05 ± 1.19 ^c^	37.96 ± 2.21 ^d^
Fe^3+^ Reducing Capability/μmol FeSO_4_·g^−1^ *PL*	438.95 ± 9.05 ^a^	440.69 ± 4.62 ^a^	417.58 ± 3.89 ^c^	383.41 ± 8.19 ^d^	378.66 ± 7.90 ^d^	427.08 ± 4.51 ^b^

Multiple comparisons by row, marked with different lower case letters indicate significant differences between groups (*p* < 0.05).

**Table 2:** Electronic nose sensor array and its performance description.

Sensors	Compound Type	Sensors	Compound Type
S1	Aromatic compounds	S8	Amines
S2	Nitrogen oxides, low molecular amines	S9	Hydrogen
S3	Sulphide	S10	Furans
S4	Organic acid esters and terpenes	S11	Volatile organic compound
S5	Terpenes, Esters	S12	Sulfide
S6	Sterols, triterpenes	S13	Vinyl
S7	Oxygenated derivatives of aliphatic hydrocarbons	S14	Lactones, pyrazines

**Table 3:** Results of Heracles NEO Ultra-Fast Gas Phase Electronic Nose measurements on samples of *PL* with different fermentation times.

NO.	Possible Compounds	CAS	Formula	Fermentation Time (Day)						Odour Characteristics
				0	1	2	3	4	5	
1	Ethanol	64-17-5	C_2_H_6_O	+	↓	↑	↑	↑	↑	Alcoholic; Spicy; Strong; Sweet
2	Methanol	67-56-1	CH_4_O	−	−	−	−	−	+	Alcohol; Spicy; Strong
3	β-Pinene	127-91-3	C_10_H_16_	+	↓	↑	−	−	−	Dry; Freshly cut grass; Pine; Resin; Sweet
4	Propanal	123-38-6	C_3_H_6_O	−	+	↑	↑	↑	−	Acetaldehyde; Cocoa; Nuts; Plastic; Spicy
5	2-Methylnonane	871-83-0	C_10_H_22_	−	−	+	−	−	−	/
6	2-Methyl-2-propanol	75-65-0	C_4_H_10_O	−	−	−	+	↑	↑	Camphor
7	(E)-2-Hexen-1-ol, butanoate	53398-83-7	C_10_H_18_O_2_	−	−	−	+	−	−	Apple; Apricot; Banana (ripe); Cheese; Fermented; Freshly cut grass
8	5-ethyl-3-hydroxy-4-methyl-2(5H)-furanone	698-10-2	C_7_H_10_O_3_	−	−	−	+	−	−	Brown Sugar; Cream Candy; Caramel; Nutty; Condiment; Spicy; Sweet
9	Tridecane	629-50-5	C_13_H_28_	−	−	−	+	−	−	Alkanes; Oranges; Fruits; Heteroalcohols
10	Carvone	6485-40-1	C_10_H_14_O	−	−	−	+	−	−	Basil; Bitter; Coriander; Fennel; Minty; Peppermint; Ruminal; Sweet
11	3-Methyl-dodecane	17312-57-1	C_13_H_28_	−	−	−	+	−	−	/
12	Anisyl alcohol	105-13-5	C_8_H_10_O_2_	−	−	−	+	−	−	Floral; Vegetative; Powdery
13	E-Tetradec-7-ene	41446-63-3	C_14_H_28_	−	−	−	+	↓	−	Freshly cut grass
14	2-Methyltetradecane	1560-95-8	C_15_H_32_	−	−	−	+	−	−	/
15	3-Ethyltridecane	13286-73-2	C_15_H_32_	−	−	−	+	−	−	/
16	3-Methyltetradecane	18435-22-8	C_15_H_32_	−	−	−	+	−	−	/
17	Pentadecane	629-62-9	C_15_H_32_	−	−	−	+	−	−	Alkanes; Heteroalcohols; Freshly cut grass
18	Propylenglycol	57-55-6	C_3_H_8_O_2_	−	−	−	−	+	−	Alcohol; Caramel; Flavourless
19	2,3-Butanediol	513-85-9	C_4_H_10_O_2_	−	−	−	−	+	−	Creamy; Fruit; Flavourless; Onion
20	Methylcyclohexane	108-87-2	C_7_H_14_	−	−	−	−	−	+	Fuzzy, Dizzy; Fruit; Sweet
21	5-Methylfurfural	620-02-0	C_6_H_6_O_2_	−	−	−	−	−	+	Acidic; Almond; Caramel; Coffee; Spicy
22	Cis-Decalin	493-01-6	C_10_H_18_	−	−	−	−	−	+	/

The presence is indicated by ‘+’, absence by ‘−’, increase in content compared to the previous day is denoted by ‘↑’, and decrease is denoted by ‘↓’.

**Table 4:** Parameters of models related to PCA and OPLS-DA analyses.

Models	PCA		OPLS-DA		
	R^2^X	Q^2^	R^2^X	R^2^Y	Q^2^
0 vs. 1	0.845	0.614	0.844	0.992	0.903
0 vs. 2	0.861	0.647	0.857	0.995	0.977
0 vs. 3	0.911	0.776	0.911	0.999	0.992
0 vs. 4	0.942	0.853	0.940	0.994	0.979
0 vs. 5	0.953	0.881	0.906	0.996	0.987

**Table 5:** Core targets in the treatment of hyperlipidaemia.

NO.	Gene	Sensors	Degree
1	EGFR	Epidermal Growth Factor Receptor	33
2	ESR1	Estrogen Receptor 1	37
3	SERPINE1	Serpin Family E Member 1	23
4	ERBB2	Erb-B2 Receptor Tyrosine Kinase 2	26
5	PPARG	Peroxisome Proliferator Activated Receptor Gamma	37
6	MMP9	Matrix Metallopeptidase 9	32
7	ACE	Angiotensin I-Converting Enzyme	29
8	HSP90AA1	Heat Shock Protein 90 Alpha Family Class A Member 1	28
9	AKT1	AKT Serine/Threonine Kinase 1	46
10	PPARA	Peroxisome Proliferator Activated Receptor Alpha	20

**Table 6:** Protein-Ligand MMPBSA analysis.

Energy	AKT1-Luteolin	MMP9-Genistein	ESR1-Daidzein
Van der Waals Energy (KJ/mol)	−141.890	−178.102	−152.691
Electrostatic energy (KJ/mol)	−32.798	−43.121	−36.634
Polar solvation energy (KJ/mol)	141.124	149.786	129.209
Nonpolar solvation Energy (KJ/mol)	−20.120	−17.975	−18.206
Total Binding Energy (KJ/mol)	−53.684	−89.412	−78.322

## Data Availability

The original contributions presented in this study are included in the article. Further inquiries can be directed to the corresponding authors.
